# Artificial selection reveals the role of transcriptional constraints in the maintenance of life history variation

**DOI:** 10.1002/evl3.166

**Published:** 2020-04-07

**Authors:** Joel L. Pick, Masaomi Hatakeyama, Kate E. Ihle, Julien Gasparini, Claudy Haussy, Satoshi Ishishita, Yoichi Matsuda, Takashi Yoshimura, Masahiro M. Kanaoka, Rie Shimizu‐Inatsugi, Kentaro K. Shimizu, Barbara Tschirren

**Affiliations:** ^1^ Department of Evolutionary Biology and Environmental Studies University of Zurich Zurich 8057 Switzerland; ^2^ School of Biological, Earth and Environmental Sciences University of New South Wales Sydney Australia; ^3^ Current Address: Institute of Evolutionary Biology University of Edinburgh Edinburgh United Kingdom; ^4^ Functional Genomics Center Zurich Zurich 8057 Switzerland; ^5^ Laboratoire Ecologie and Evolution UMR 7625 Université Pierre et Marie Curie CNRS ENS Paris France; ^6^ Graduate School of Bioagricultural Sciences Nagoya University Nagoya 464–8602 Japan; ^7^ Graduate School of Science Nagoya University Nagoya 464–8601 Japan; ^8^ Kihara Institute for Biological Research Yokohama City University Yokohama 244–0813 Japan; ^9^ Centre for Ecology and Conservation University of Exeter Penryn TR10 9FE United Kingdom

**Keywords:** Costs of reproduction, egg size, immune defence, life‐history trade‐offs, maternal care, natural selection

## Abstract

The trade‐off between reproduction and self‐maintenance is a cornerstone of life history theory, yet its proximate underpinnings are elusive. Here, we used an artificial selection approach to create replicated lines of Japanese quail (*Coturnix japonica*) that differ genetically in their reproductive investment. Whole transcriptome sequencing revealed that females from lines selected for high reproductive output show a consistent upregulation of genes associated with reproduction but a simultaneous downregulation of immune genes. Concordant phenotypic differences in immune function (i.e., specific antibody response against keyhole limpet hemocyanin) were observed between the selection lines, even in males who do not provide parental care. Our findings demonstrate the key role of obligate transcriptional constraints in the maintenance of life history variation. These constraints set fundamental limits to productivity and health in natural and domestic animal populations.

**Impact Summary**

A central tenet in life history theory is that key functions, such as reproduction and self‐maintenance, trade‐off against each other, preventing organisms from expressing perfect phenotypes. However, surprisingly little is known about the mechanisms and pathways underlying such fundamental trade‐offs. Our experiments demonstrate that female birds artificially selected for high reproductive investment upregulate expression of genes associated with reproduction, but simultaneously downregulate genes related to immune function. Immunosuppression at the phenotypic level was also observed in both sexes, despite males not contributing to parental care, and in the absence of resource limitation, demonstrating its obligate nature. By linking evolutionary theory with mechanistic function, our study reveals that intrinsic transcriptional constraints act to maintain diversity in life histories, and set limits to productivity and health in natural and domesticated populations.

Given that greater investment in reproduction results in more and higher quality offspring (McGinley et al. 1987; Fox and Czesak 2000; Krist 2011), natural selection should favor high reproductive efforts. Yet, within and across taxa remarkable variation in how much individuals invest in reproduction is observed. This variation is believed to be maintained through trade‐offs that prevent individuals from investing maximally in all fitness‐related traits and functions simultaneously (Williams 1966; van Noordwijk and de Jong 1986; Stearns 1992, 1989). An increase in reproductive investment may, therefore, be accompanied by a simultaneous decrease in somatic maintenance (e.g., in immune function or stress tolerance). However, despite their fundamental role in life history evolution, and decades of research into their ecological and evolutionary consequences, little is known about the proximate mechanisms that underlie life history trade‐offs (Zera and Harshman 2001; Flatt and Heyland 2011; Schwenke et al. 2016). Furthermore, whether such trade‐offs are resource dependent (as traditionally thought) or occur in the absence of resource limitation (i.e., are obligate) is still poorly understood (Hosken 2001; Leroi 2001; Barnes and Partridge 2003; Harshman and Zera 2006; Stahlschmidt et al. 2013). Genomic approaches, in combination with experimental manipulations of life history strategies, promise novel insights into the mechanisms and pathways underlying life history evolution and the maintenance of diversity in life histories within and across populations (Stearns and Magwene 2003; Roff 2007). Such approaches benefit from having no a priori assumptions about the nature of the mechanisms involved, and so represent an unbiased view of the trade‐offs and constraints that contribute to and maintain diversity in life histories.

In oviparous species, variation in parental investment occurs primarily at the prenatal stage, in the form of variation in the quantity of resources a mother provides to her offspring through the egg. Given that egg size has a strong positive effect on offspring fitness across taxa (McGinley et al. 1987; Fox and Czesak 2000; Krist 2011), we may expect mothers to invest maximally in their eggs. However, a large amount of variation in egg size is commonly observed within and across populations (e.g., Christians 2002). To unravel the trade‐offs and constraints that maintain diversity in reproductive strategies, we used a whole transcriptome sequencing approach in a population of Japanese quail (*Coturnix japonica*) artificially selected for divergent reproductive investment (Pick et al. 2016b).

Previously, we observed a strong divergence in egg mass between the replicated lines after only a few generations of directional selection, but no correlated response in the number of eggs laid (Pick et al. 2016b). Females from the divergent selection lines therefore differ substantially in the total investment they make into reproduction. Variation in egg size is largely a function of variation in the size of the yolk (Williams et al. 2001), which contains all the fat and approximately half the protein the mother provides for the developing young (Carey et al. 1980). Accordingly, our selection regime significantly affected yolk size, as well as the dry constituents of yolk (Pick et al. 2016b). Because the divergent lines do not differ in the rate of egg laying (Pick et al. 2016b), differences in egg size between selection regimes must be due to different growth rates of developing ovarian yolk follicles. In line with this, correlative evidence in zebra finches (*Taeniopygia guttata*) has shown that uptake of nutrients into the developing yolk follicle is greater in females laying larger eggs (Han et al. 2009). As the developing follicles are the site at which differential maternal resource investment physically occurs, follicles in the rapid growth phase are prime sites to detect trade‐offs and constraints.

By using a whole transcriptome sequencing approach to identify the molecular mechanisms and transcriptional pathways underlying fundamental life history trade‐offs, we demonstrate that genes associated with reproduction are consistently upregulated in females selected for increased reproductive effort, whereas genes associated with immune function are consistently downregulated. This reproduction‐immunity trade‐off is corroborated at the phenotypic level, in both sexes and in the absence of resource limitation, demonstrating the intrinsic and obligate nature of this trade‐off.

## Methods

### EXPERIMENTAL DESIGN

We established replicated, divergent artificial selection lines for high (H) and low (L) maternal egg investment in a population of Japanese quail (Pick et al. 2016b). Briefly, the top 25% of females producing the largest and smallest eggs relative to their body size were bred to establish the high and low investment lines, respectively. This procedure was performed with two independent groups of birds to create two independent biological replicates (H1/L1 and H2/L2). In subsequent generations, the 50% of females with the most extreme phenotype within each line and replicate were selected to continue the selection regime. The high and low investment lines within a replicate were bred simultaneously, meaning that within a replicate birds from the H and L lines encountered the same environmental conditions and were of the same age. All birds had access to ad libitum food at all times (i.e., they were not resource limited). Levels of inbreeding within each line replicate were negligible (see Pick et al. 2016b). All procedures complied with all relevant ethical regulations and were conducted under licenses provided by the Veterinary Office of the Canton of Zurich, Switzerland (permits 195/2010, 14/2014, 156).

### DISSECTION AND TISSUE COLLECTION

F3 follicles from 55 females (H1: *N* = 12; H2: *N* = 19; L1: *N* = 13; L2: *N* = 11) from the fourth generation of the selection experiment were collected for this study. The F3 follicle was chosen because it is one of the follicles in the rapid follicle growth phase and shows high levels of gene expression (Han et al. 2009).

The females were kept in single sex pairs in breeding cages prior to dissections. The day before dissection, individual laying times were recorded and females were dissected approximately 18 hours after laying, so the stage of egg production was standardized among females. For tissue collection, animals were first stunned and then euthanized via exsanguination. F3 follicles were immediately harvested and weighed, the yolk was removed from the follicle, and the remaining follicle tissue was washed in saline solution, before being flash frozen in liquid nitrogen. Total time from animal capture to follicle flash freezing was less than 10 minutes (range: 6–10 minutes) for each sample. Samples were stored at –80°C until extraction. The remaining yolky follicles (F1, F2, and F4) and yolk of the oviductal egg were subsequently dissected out and weighed to test for differences in follicular growth rates between the selection lines (see below).

### RNA EXTRACTION

We extracted RNA from F3 follicles of 20 females (five females from each of the four line replicates, H1, L1, H2, and L2, matched as far as possible for body mass) for RNA‐seq and targeted quantitative PCR (qPCR). Follicle tissue was thoroughly homogenized, and RNA was extracted using TRizol Reagent (Invitrogen, Switzerland) and purified using the RNeasy kit (Qiagen, Switzerland). We quantified RNA concentrations with Qubit (Invitrogen, Switzerland).

### RNA‐seq

A total of 16 samples (four nonsibling females from each line replicate: H1, L1, H2, and L2) were submitted to the Functional Genomics Center Zurich for library preparation and sequencing (RNA‐seq). Libraries were created from 1 μg of RNA using the TruSeq RNA Stranded kit (Illumina, USA). Samples were then sequenced on the HiSeq2500 platform (Illumina, USA; 1 × 125 bp) in a single lane. Sequence data were submitted to DDBJ (BioProject Submission ID: PRJEB11185, BioSample Submission ID: SAMEA3578958–SAMEA3578974). The RNA‐seq data analysis pipeline described below was implemented and executed on the SUSHI NGS analysis framework (Hatakeyama et al. 2016) at the Functional Genomics Center Zurich. Sequence quality was checked using FastQC version 0.11.3 (Andrews 2010). Adapters and low‐quality bases were trimmed using Trimmomatic 0.33 (Bolger et al. 2014) before read alignment. A typical count‐based differential gene expression analysis was conducted by using STAR 2.3.1b (Dobin et al. 2013) for reads alignment, Rsubread 1.22.2 (Liao et al. 2013) for mapped read counting, and edgeR 3.14.0 (Robinson et al. 2009) for detection of differentially expressed genes (DEGs) between selection regimes in R 3.3.0. The count data were normalized by the trimmed means of M‐values method and a generalised linear model (GLM)‐based likelihood ratio test was used to infer statistical significance (*P* < 0.05) of the selection regime effect. Initial quality checks revealed that four samples (one sample from each line replicate) did not cluster with the others and they were therefore excluded from downstream analyses.

We used the chicken (*Gallus gallus*) genome (Ensembl release 84) as a reference genome. In addition, we performed the same analyses using two genome assemblies of the Japanese quail: Coturnix japonica 2.0 (GenBank assembly accession: GCA_001577835.1) and Coja_2.0a (GenBank assembly accession: GCA_000511605.2, upgraded from the assembly reported by Kawahara‐Miki et al. [2013]). As the chicken genome has the most complete assembly and is better annotated, we used it as our primary reference. Similar expression patterns were observed when using the *C. japonica* genomes (Figure S1 and Tables S1–S3). We observed a large overlap in the genes identified with each reference genome, lending support to the robustness of the analysis with *G. gallus* as a reference genome: 50.8% (229 genes) were identified as consistently differentially expressed across the three analyses (Figure S2 and Tables S1–S3). However, as both quail genomes rely heavily on the chicken genome for assembly and annotation, the agreement between results is expected.

We first tested for broad differences in gene expression between the selection regimes across both replicates and performed a gene ontology (GO) enrichment analysis on the total set of genes that were differentially expressed between the high and low investment lines. Annotation of the genes based on the chicken genome was performed using homology searching of coding region by NCBI Blast 2.2.31 (Madden 2013). We used topGO 2.26.0 with elim algorithm (Alexa and Rahnenfuhrer 2016) in R 3.3.0, with a significance level of *P* < 0.01 on Biological Processes.

Second, to account for founder effects and drift that occurs in small populations (Falconer and Mackay 1996), which can lead to differential gene expression by chance, as well as the false positive discovery of DEGs, we used a more conservative approach, in which we analyzed the two replicates separately, and only considered genes that were consistently significantly differentially expressed between the selection regimes in both independent replicates.

### TARGETED qPCR

To verify the RNA‐seq results, we selected seven genes with known gene function that were consistently differentially expressed between the high and low reproductive investment selection regimes across replicates (genes related to reproduction: *VTG2*, *NELL2*, *KIAA1211*, and *ADAMTS18*; genes related to immune function: *TLR3*, *ASPN*, and *Mx*; see Results), and measured levels of gene expression for these genes using quantitative real‐time PCR (qPCR) in a larger number of individuals from the divergent lines. We converted RNA samples from the 20 individuals described above to cDNA using the High Capacity RNA‐to‐cDNA kit (Invitrogen, Switzerland) according to the manufacturer's instructions. Primers were designed based on the *C. japonica* genome (Table S8) and validated via BLAST search, gel electrophoresis, and meltcurve analyses. We determined primer efficiencies via standard curve analysis. One‐step qPCR was performed on a OneStepPlus system using the SybrSelect reagent (Applied Biosystems, USA). Samples were run in triplicate and analyzed relative to expression of the housekeeping gene β‐actin. We calculated the relative expression of target genes following the method of (Pfaffl 2001). For all genes, one or two samples did not fulfill quality requirements, resulting in a sample size of *N* = 18 or 19 across genes. We tested for differences in qPCR‐based gene expression between selection regimes using linear models in R 3.3.0, including selection regime (H vs. L) and replicate (1 vs. 2) as fixed factors.

### PHENOTYPIC DIFFERENCES IN REPRODUCTION AND IMMUNE RESPONSE

To further consolidate the observed patterns, we verified the transcriptional differences in reproductive investment and immune function between the selection regimes (see Results) with phenotypic measures in birds from the selection experiment. First, we tested if yolk deposition (and so maternal resource investment) occurred at a faster rate in high investment females. To this end, we analyzed differences in follicle growth rate between the selection regimes using random regression models, using the weights of the yolky follicles (F1–F4) and yolk of the oviductal egg of the 55 dissected females (see above). Assuming that all individuals ovulate at 24 hours intervals (Pick et al. 2016b), we could assign each follicle an approximate time before ovulation in hours (yolk: 0, F1: –6, F2: –30, F3: –54, F4: –78). To linearize the relationship between follicle size and time before ovulation, we took the square‐root of follicle mass as the dependent variable. Time before ovulation (transformed to days for analysis) and body size (measured as tarsus length [in mm]) were included as covariates and selection regime and replicate as fixed factors. We also included the interaction between time before ovulation and selection regime to test whether follicle growth rate differed between the divergent selection lines. Female identity was included as a random effect, with time before ovulation being allowed to vary within each female (random slopes). Significance of fixed effects was determined by comparing nested models (with and without the variable of interest) using likelihood ratio tests. Models were run in R 3.3.0 using the lme4 package (1.1‐12).

Second, we paired 80 adult females (42 H line and 38 L line) with 80 adult males (40 H line and 40 L line) from generations six and seven of the selection experiment and housed them in individual breeding cages for 14 days. Eggs were collected and weighed each day. All birds were in reproductive condition, except for four females who were excluded from further analyses (age of birds: 182–210 days old). After the quantification of the female's reproductive investment, we immune challenged these birds with keyhole limpet hemocyanin (KLH) to measure their specific antibody response against a novel antigen. This approach was chosen for both practical and ethical reasons. Although it allows us to measure the humoral immune response against a novel antigen, the measured response may not be directly comparable to the birds’ response against live pathogens encountered in the wild. We used birds from later generations for phenotypic measures of immune response because differences in immune gene expression between the selection regimes were only known after the expression analysis (see above) was completed. Given that a standardized selection regime was applied throughout the selection experiment, the transcriptional differences observed in generation 4 were likely similar or even stronger in these later generations.

We took an initial blood sample from the brachial vein using heparinized capillary tubes, injected individuals subcutaneously with 50 μg of KLH in 50 μL PBS, and took a second blood sample one week postimmunization, at the peak of the anti‐KLH antibody response in quail (unpublished data). Samples were kept on ice until centrifugation (5 min at 20°C and 2000 × *g*). Plasma was then separated and frozen at –80°C until analysis. Anti‐KLH antibody levels in the plasma were determined using a sandwich ELISA technique following a modified protocol described in Jacquin et al. (2013) (and in further detail in the Supporting Information). This resulted in a measure of the relative antibody concentration (Ab), which was log transformed for further analysis. We calculated the difference between the postinjection Ab and the preinjection baseline Ab as our measure of immune response (delta Ab). For five birds (two males and three females), we did not get a blood sample both before and after the immune challenge, and so they were excluded from further analysis. This left data for 73 females (36 H line and 37 L line) and 78 males (38 H line and 40 L line). We analyzed delta Ab using a linear mixed model, including selection regime, sex, replicate, and generation, as well as the interaction between selection regime and sex. This interaction was removed from the final model as it was not significant. As the sample of birds for which antibody response was measured included siblings, maternal ID was added as a random effect to account for nonindependence.

To demonstrate the continued difference in reproductive investment between the divergent selection lines in these later generations, we used the same model structure as above, but without sex and the selection regime × sex interaction, to test for a difference in egg size between the females in this sample (*N* = 73). Significance of fixed effects in both models was determined by comparing nested models (with and without the variable of interest) using likelihood ratio tests. Models were run in R 3.3.0 using the lme4 package (1.1‐12). All statistical tests performed in this study were two sided.

## Results

### DIFFERENCES IN TRANSCRIPTOME‐WIDE GENE EXPRESSION BETWEEN LINES SELECTED FOR DIVERGENT REPRODUCTIVE INVESTMENT

We analyzed transcriptome‐wide gene expression levels in the rapidly growing F3 ovarian follicle of 12 females from replicated lines artificially selected for either high or low maternal egg investment (three from each of the four line replicates), with a sequencing coverage of 26–43 million reads per sample. Examining broad differences in gene expression across all females revealed 346 genes that were significantly differentially expressed between the divergent lines, 180 of which were upregulated in the high investment lines relative to the low investment lines, whereas 166 were downregulated (Table S1). GO enrichment analysis of these DEGs identified 58 overrepresented GO categories (Table S4), including diverse GO categories associated with immune function, such as response to viruses (GO:0009615), negative regulation of viral genome replication (GO:0045071), interferon‐gamma‐mediated signaling pathway(GO:0060333), MyD88‐independent toll‐like receptor signaling pathway (GO:0002756), regulation of interferon‐beta biosynthetic process (GO:0045357), positive regulation of production of molecular mediators of immune response (GO:0002702), positive regulation of interleukin‐8 production (GO:0032757), regulation of cytokine production involved in immune response (GO:0002718), and regulation of cytokine‐mediated signaling pathway (GO:0001959; for full list, see Table S4).

When using a more conservative approach that only considered genes that were significantly differentially expressed between the divergent selection regimes in both independent replicates, we identified 66 DEGs. However, for 36 of these genes, the direction of expression difference was not consistent between the two replicates, resulting in a final set of 30 genes that were *consistently* differentially expressed between the high and low investment lines in both independent biological replicates (Table 1). All of these consistently DEGs were also present in the full list of 346 DEGs (Table S1).

**Table 1:** Description and broad function of the 30 genes that were consistently differentially expressed between the high and low reproductive investment lines in both independent biological replicates. Expression differences for the two replicates are presented separately

			Replicate 1		Replicate 2		
Gene name	Description	Function (Reference)	Log2 ratio	*P*‐value	Log2 ratio	*P*‐value	Ensembl GID
**L > H**							
*ADSSL1*	Regulation of purine nucleotide cycle, muscle metabolism	Metabolism (Sun et al. 2005)	−0.604	0.005	−0.572	0.011	ENSGALG00000011618
*ASPN*	Innate immunity	Immunity (Ng et al. 2011)	−0.708	0.031	−0.975	0.000	ENSGALG00000004722
*COL28A1*	Immune response	Immunity (Kennedy et al. 2012; Boon et al. 2014)	−1.700	0.047	−1.991	0.000	ENSGALG00000010259
*EXO1*	DNA repair	DNA repair (Nebel et al. 2009; Mason and Cox 2012; Tomimatsu et al. 2012)	−0.540	0.031	−0.830	0.001	ENSGALG00000010748
*Mx*	Viral defense, avian influenza resistance	Immunity (Ewald et al. 2011; Sartika et al. 2011)	−1.061	0.040	−1.120	0.000	ENSGALG00000016142
*PATL2*	RNA binding and decay	Gene expression (Ozgur et al. 2010)	−0.795	0.036	−0.702	0.027	ENSGALG00000002167
*PPP4R4*	Protein phosphatase regulation, NF‐κB activation	Immunity (Gewurz et al. 2012)	−0.996	0.030	−0.781	0.013	ENSGALG00000010950
*TLR3*	Virus detection, activation of immune response	Immunity (Alexopoulou et al. 2001; Cheng et al. 2014)	−0.624	0.006	−0.419	0.017	ENSGALG00000013468
*USH1G*	Development of the auditory and visual systems	Vision (Yan and Liu 2010)	−1.467	0.038	−1.587	0.033	ENSGALG00000007775
Unknown	–	–	−0.958	0.008	−1.612	0.001	ENSGALG00000019233
Unknown	–	–	−3.485	0.000	−2.342	0.013	ENSGALG00000013110
**H > L**							
*7SK*	RNA polymerase promotion	Gene expression (Bannister et al. 2007)	1.280	0.019	1.288	0.011	ENSGALG00000017926
*ADAMTS18*	Follicle development, ovulation	Reproduction (Ataca et al. 2016)	1.528	0.020	1.290	0.015	ENSGALG00000005347
*CCDC64*	Neurohypophyseal hormone activity	Reproduction (Guan et al. 2016)	1.318	0.048	1.726	0.013	ENSGALG00000007319
*COL8A2*	Membrane formation	Vision (Gottsch et al. 2005)	1.012	0.001	0.855	0.007	ENSGALG00000002298
*IGSF3*	Placental function	Reproduction (Goyal et al. 2010)	0.697	0.023	0.420	0.030	ENSGALG00000015469
*KIAA1211*	Very low density lipoprotein (VLDL) synthesis	Reproduction, body fat (Wu et al. 2016)	1.167	0.020	0.803	0.001	ENSGALG00000013771
*NEGR1*	Neuronal growth regulator, control of body mass	Neural function, body mass (Lee et al. 2012)	1.635	0.035	1.540	0.004	ENSGALG00000011350
*NELL2*	Regulation of estrous cycle	Reproduction (Ryu et al. 2011)	1.044	0.003	0.904	0.000	ENSGALG00000009601
*PROM1*	Cell proliferation, binding of cholesterol	Vision (Zacchigna et al. 2009)	0.691	0.032	1.180	0.000	ENSGALG00000014496
*QPCT*	Regulation of gonadotropin—releasing hormone and estrogen levels	Reproduction (Ezura et al. 2004)	0.506	0.015	0.489	0.017	ENSGALG00000010617
*RARRES1*	Retinoid metabolism	Vision (Zaitseva et al. 2008)	0.681	0.001	0.468	0.033	ENSGALG00000009594
*RIMS1*	Regulation of synaptic vesicle exocytosis and calcium channels	Metabolism (Gandini et al. 2011)	1.500	0.001	0.877	0.010	ENSGALG00000015944
*SYNDIG1L*	Synapse differentiation	Reproduction (Verardo et al. 2016)	1.924	0.035	1.785	0.012	ENSGALG00000010233
*VTG2*	Yolk synthesis	Reproduction (Deeley et al. 1975)	2.002	0.042	1.392	0.031	ENSGALG00000001863
*ZPD*	Oocyte formation	Reproduction (Okumura et al. 2004)	1.243	0.034	0.986	0.006	ENSGALG00000001069
Unknown	–	–	1.089	0.000	0.519	0.010	ENSGALG00000026592
Unknown	–	–	1.278	0.000	0.663	0.006	ENSGALG00000028371
Unknown	–	–	1.363	0.002	1.677	0.000	ENSGALG00000025825
Unknown	–	–	1.439	0.036	1.269	0.044	ENSGALG00000028038

As expected, many of the genes that were consistently upregulated in the high investment lines relative to the low investment lines (*N* = 19) were associated with reproductive functions, such as yolk synthesis (*VTG2*), follicle development (*ADAMTS18* and *ZPD*) or the regulation of the oestrous cycle (*NELL2*, *QPCT*, and *CCDC64*) (Table 1). Strikingly, among the genes (*N* = 11) that were consistently downregulated in the high investment lines relative to the low investment lines, many were associated with self‐maintenance (*Exo1*), and with immune function in particular (*ASPN*, *COL28A1*, *Mx*, *PPP4R4*, and *TLR3*; Table 1). For five of the 30 consistently DEGs, the gene function was unknown (Table 1).

### TARGETED qPCR OF CANDIDATE GENES IDENTIFIED USING RNA‐seq

**Figure 1:**
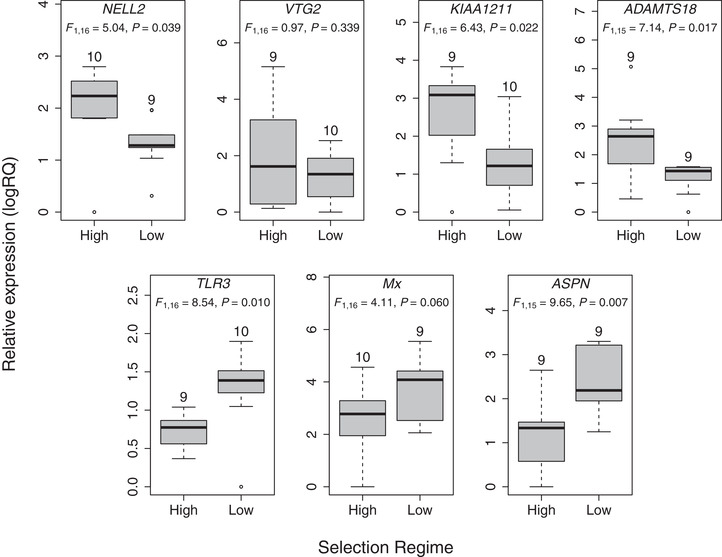
Differences in the expression of genes related to reproduction (*NELL2*, *VTG2*, *KIAA1211*, and *ADAMS18*) and immune function (*TLR3*, *Mx*, and *ASPN*) between lines artificially selected for high or low reproductive investment. Candidate genes were identified with whole transcriptome sequencing and verified using qPCR. Results of linear models testing for expression differences based on qPCR between the selection regimes are shown. In boxplots, the center line shows the median; box limits show upper and lower quartiles; whiskers show 1.5× interquartile range; points show outliers. Sample sizes are given above each boxplot.

We verified expression patterns in a subset of consistently DEGs with known gene function (see above) in a larger set of individuals (*N* = 18 or 19) using qPCR. Expression patterns of all these genes were consistent with the RNA‐seq results (marginally nonsignificant for *Mx*), with the exception of *VTG2* (Fig. 1; Table S5). Note, however, that *VTG2* had very low expression levels overall (Table S1), reducing the power to detect differences.

### PHENOTYPIC DIFFERENCES IN REPRODUCTIVE INVESTMENT AND SPECIFIC ANTIBODY RESPONSE

**Figure 2:**
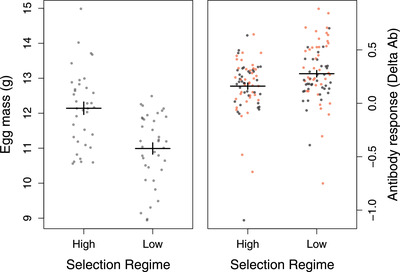
Phenotypic differences in reproductive investment and immune response between lines artificially selected for high or low reproductive investment. Immune response was measured as the specific antibody response against a novel antigen (KLH) (difference between pre‐ and postimmunization antibody levels; Delta Ab). Means and standard errors are shown as horizontal and vertical bars, respectively. Raw data are shown; females in black and males in red.

In line with the patterns observed at the transcriptional level, we observed a pronounced difference in reproductive investment between the divergent selection regimes at the phenotypic level with females from the high investment lines showing significantly faster ovarian follicle growth rates (*N* = 55, *χ* ^2^ = 11.91, *P* < 0.001; Table S6) and laying significantly larger eggs (*χ* ^2^ = 19.46, *P* < 0.001; Fig. 2; Table S7). At the same time, individuals from the high investment lines had a significantly lower specific antibody response when challenged with a novel antigen (KLH) than individuals from the low investment lines (*χ* ^2^ = 5.47, *P* = 0.019; Fig. 2; Table S7). Importantly, this effect did not differ between males and females (sex × line interaction: *χ* ^2^ = 2.01, *P* = 0.156; Table S7).

## Discussion

Here, we demonstrate that artificial selection for increased reproductive investment in a precocial bird results in an upregulation of genes associated with reproductive function but a simultaneous downregulation of immune genes (and vice versa in birds selected for decreased reproductive investment), across independent biological replicates. These findings complement previous work that has demonstrated genetic and physiological trade‐offs between reproductive effort and immune function in various taxa—most notably in *Caenorhabditis elegans* (Leroi 2001; Barnes and Partridge 2003; Miyata et al. 2008) and insects (predominantly *Drosophila*; Schwenke et al. 2016; Fabian et al. 2018). In vertebrates, experimental work has linked postnatal parental effort to immune phenotypes (Knowles et al. 2009) and more generally reproductive hormones (e.g., testosterone) to immune phenotypes in several taxa (Foo et al. 2017; but see Roberts et al. 2004; Martin et al. 2008). Our experimental results add to this literature by highlighting the key role of transcriptional constraints in mediating life history trade‐offs and contributing to the maintenance of diversity in reproductive strategies in the natural world.

Traditionally, life history trade‐offs have been assumed to be the result of an internal struggle for limited resources (Williams 1966; van Noordwijk and de Jong 1986; Stearns 1992, 1989). However, more recent studies investigating the physiological regulation of life history trade‐offs have suggested that they may be obligate (Hosken 2001; Leroi 2001; Barnes and Partridge 2003; Harshman and Zera 2006; Flatt and Heyland 2011; Stahlschmidt et al. 2013), caused either by genetic correlation (e.g., through pleiotropy; Zhong et al. 2005) or by physiological damage caused by reproductive function that directly impairs self‐maintenance (Tatar and Carey 1995). Because a reduced KLH‐specific antibody response was not only observed in females of the high reproductive investment lines, but also in males, who do not contribute to offspring provisioning, it is unlikely that direct damage induced by increased reproductive effort (e.g., damage caused by oxidative stress; Alonso‐Alvarez et al. 2004) underlies the observed reduction in immune function (see also Pick et al. 2016a). Furthermore, birds in our study had access to ad libitum food at all times, which makes it unlikely that the observed trade‐off between reproductive investment and immune function was mediated by resource limitation. Rather it suggests that obligate intrinsic constraints, such as regulatory constraints in transcriptional networks or cascades (i.e., shared trans regulatory elements), may underlie its occurrence. Such constraints will act to limit the evolution of both parental care and pathogen defense, and contribute to the maintenance of diversity in reproductive strategies in the natural world. Identifying the mechanisms driving such transcriptional constraints will be an exciting next step. Furthermore, a manipulation of resource availability and subsequent quantification of the effects on patterns of gene expression in birds from the divergently selected lines would provide further insight into the relative importance of, and potential interactions among, external and internal factors in mediating the observed trade‐offs.

Not only are these findings relevant for our understanding of life history evolution, but they also have profound implications for the animal breeding industry. Breeding programs universally aim at increasing reproductive output, and our results suggests that, as an unavoidable consequence, they will simultaneously select for poorer health. Indeed, many commercial chicken breeds are known to be particularly susceptible to disease (Jie and Liu 2011). In this study, many of the genes that were consistently downregulated in birds artificially selected for increased reproductive investment were associated with immune function. For example, Myxovirus resistance‐1 (*Mx1*) has been directly linked to resistance against highly pathogenic strains of avian influenza in poultry (Ewald et al. 2011), *Col28a1* is associated with influenza virus resistance in mice (Boon et al. 2014), and Toll‐like receptor 3 (*Tlr3*) has been associated with the response to Newcastle disease virus (Cheng et al. 2014). Together with the results of the GO enrichment analysis, these findings suggest that the response to viruses in particular is impaired in animals that have been selected for increased reproductive effort. In addition to immunity‐related genes, we observed a consistent downregulation of Exonuclease 1 (*Exo1*) in birds selected for increased reproductive investment. *Exo1* encodes a protein that plays an essential role in DNA repair mechanisms and the maintenance of genome stability (Mason and Cox 2012), and increased *Exo1* expression has been linked to longevity in humans (Nebel et al. 2009). Together, these transcriptional responses to our selection regime, observed in two independent biological replicates, suggest that reproduction, longevity/ageing, and aspects of immune function are intimately interlinked, and that resulting “life history syndromes” are readily shaped by natural or artificial selection.

In conclusion, our study provides experimental evidence for a key role of transcriptional constraints in mediating life history trade‐offs, and suggests that intrinsic constraints in transcriptional networks or cascades contribute to the maintenance of variation in parental care and immune defense. Understanding the nature of these transcriptional constraints may open up new avenues to increase productivity and health in natural and domestic animal populations.

## CONFLICT OF INTEREST

The authors declare no conflict of interest.

Associate Editor: A. K. Khila

## Supporting information


**Table S1**. Analysis of differential gene expression in high versus low investment lines using the chicken (*Gallus gallus*) genome as a reference.
**Table S2**. Analysis of differential gene expression in high versus low investment lines using the Japanese quail (*Coturnix japonica*) genome, Coja_2.0a (GenBank assembly accession: GCA_000511605.2, submitted by Tokyo University of Agriculture, Japan) as a reference.
**Table S3**. Analysis of differential gene expression in high versus low investment lines using the Japanese quail (*Coturnix japonica*) genome, Coturnix japonica 2.0 (GenBank assembly accession: GCA_001577835.1, submitted by Washington University School of Medicine, US) as a reference.
**Table S4**. Results of gene ontology analysis of the 346 differentially expressed genes.
**Table S5**. Differences in the expression level of seven candidate genes of known function identified using RNAseq.
**Table S6**. Differences in follicle growth rate between the selection regimes.
**Table S7**. Differences in egg size and specific antibody response against a novel antigen (KLH) between the selection regimes in generation six and seven.
**Table S8**. Primers used for targeted quantitative PCR
**Figure S1**.Mean levels of gene expression (Transcripts Per Millions) between the high and low investment lines based on the A) chicken genome (*Gallus gallus*, Ensemble release 84), B) Coturnix japonica 2.0, C) Coja_2.0a. Each point corresponds to one gene.
**Figure S2**. Venn diagram showing the overlap of differentially expressed genes between different reference genomes.

Supporting Information

Supporting Information

Supporting Information

Supporting Information
