# Supplementary material for: Artificial selection reveals the role of transcriptional constraints in the maintenance of life history variation
Source: Evol Lett. 2020 Apr 7;4(3):200–11. doi: 10.1002/evl3.166 (PMC7293072; doi:10.1002/evl3.166)
Supplement: Supplementary file 1 — Table S1. Analysis of differential gene expression in high versus low investment lines using the chicken (Gallus gallus) genome as a reference. Table S2. Analysis of differential gene expression in high versus low investment lines using the Japanese quail (Coturnix japonica) genome, Coja_2.0a (GenBank assembly accession: GCA_000511605.2, submitted by Tokyo University of Agriculture, Japan) as a reference. Table S3. Analysis of differential gene expression in high versus low investment lines using the Japanese quail (Coturnix japonica) genome, Coturnix japonica 2.0 (GenBank assembly accession: GCA_001577835.1, submitted by Washington University School of Medicine, US) as a reference. Table S4. Results of gene ontology analysis of the 346 differentially expressed genes. Table S5. Differences in the expression level of seven candidate genes of known function identified using RNAseq. Table S6. Differences in follicle growth rate between the selection regimes. Table S7. Differences in egg size and specific antibody response against a novel antigen (KLH) between the selection regimes in generation six and seven. Table S8. Primers used for targeted quantitative PCR Figure S1.Mean levels of gene expression (Transcripts Per Millions) between the high and low investment lines based on the A) chicken genome (Gallus gallus, Ensemble release 84), B) Coturnix japonica 2.0, C) Coja_2.0a. Each point corresponds to one gene. Figure S2. Venn diagram showing the overlap of differentially expressed genes between different reference genomes. [file EVL3-4-200-s001.docx]

Supplementary Materials for

**Artificial selection reveals the role of transcriptional constraints in the maintenance of life history variation**

Joel L. Pick*, Masaomi Hatakeyama, Kate E. Ihle, Julien Gasparini, Claudy Haussy, Satoshi Ishishita, Yoichi Matsuda, Takashi Yoshimura, Masahiro M. Kanaoka, Rie Shimizu-Inatsugi, Kentaro K. Shimizu & Barbara Tschirren

* joel.l.pick@gmail.com

**This file includes:**

Supplementary Methods

Supplementary Tables S1 to S8

Supplementary Figures S1 to S2

Supplementary References

**Supplementary Methods**

*Plasma anti-KLH antibody levels*

Anti-KLH antibody levels in the plasma were determined using a sandwich ELISA technique. High-binding plates (96 wells, flat bottom, Microlon 600; cat. 655101, Greiner Bio-One, Germany) were coated overnight at 4°C with 100 μl KLH (40 μg / ml in 50 mM carbonate/bicarbonate buffer, pH 9.6), then washed three times with phosphate buffered saline (PBS). Wells were blocked with 200 μl 5% milk powder (Regilait Bio) in PBS for two hours at room temperature. After washing, 100 μl of the quail plasma diluted 1:200 in PBS with 0.5% milk powder were added. Plates were then incubated overnight at 4°C. After washing, plates were incubated for two hours at room temperature with 100 μl of Goat anti-Bird IgG (H + L) (antibodies-online, n°ABIN638164) conjugated to horseradish peroxidase (1 mg / ml in PBS with 0.2% and 0.1% Pro-Clean 400), diluted 1:5000 in PBS with 0.5% milk powder. After washing, 100 μl of TMB peroxidase substrate (Bio-Rad, UK) were added to the wells. The reaction was stopped after 30 minutes by adding 50 μl HCl (1M). Plates were then read at 450 nm in a microplate reader (iMark, Bio-Rad, UK). As a standard, a mixture of several plasma samples was measured in serial dilutions to calculate a relative antibody concentration after calibrations with this standard. This relative antibody concentration (Ab), which was log transformed for further analysis. After calibration, a subset of samples was used to estimate the inter-plate (N = 9 on three different plates, *F_8,18_* = 49.42, *P* < 0.001, *r* = 0.94) and intra-plate repeatability (N = 16, *F_15,16_* = 21.3, *P* < 0.001, *r* = 0.89). The high values of repeatability show that the method is reliable to measure anti-KLH antibody concentrations.

**Supplementary Tables**

**Table S1. (separate file)**

Analysis of differential gene expression in high versus low investment lines using the chicken (*Gallus gallus*) genome as a reference.

**Table S2.** **(separate file)**

Analysis of differential gene expression in high versus low investment lines using the Japanese quail (*Coturnix japonica*) genome, Coja_2.0a (GenBank assembly accession: GCA_000511605.2, submitted by Tokyo University of Agriculture, Japan) as a reference.

**Table S3.** **(separate file)**

Analysis of differential gene expression in high versus low investment lines using the Japanese quail (*Coturnix japonica*) genome, Coturnix japonica 2.0 (GenBank assembly accession: GCA_001577835.1, submitted by Washington University School of Medicine, US) as a reference.

**Table S4. (separate file)**

Results of gene ontology analysis of the 346 differentially expressed genes.

**Table S5.**

Differences in the expression level of seven candidate genes of known function identified using RNAseq. Expression levels were quantified using qPCR. Statistically significant effects are highlighted in bold.

| Gene | Predictor | Estimate | SE | *F* | DF | *P* |
| --- | --- | --- | --- | --- | --- | --- |
| *NELL2* | Intercept | 2.089 | 0.265 |  |  |  |
|  | **Selection Regime (L)** | **-0.700** | **0.312** | **5.04** | **1, 16** | **0.039** |
|  | Replicate (2) | -0.135 | 0.312 | 0.19 | 1, 16 | 0.670 |
| *VTG2* | Intercept | 2.138 | 0.541 |  |  |  |
|  | Selection Regime (L) | -0.627 | 0.636 | 0.97 | 1, 16 | 0.339 |
|  | Replicate (2) | -0.401 | 0.636 | 0.40 | 1, 16 | 0.537 |
| *KIAA1211* | Intercept | 2.575 | 0.419 |  |  |  |
|  | **Selection Regime (L)** | **-1.250** | **0.493** | **6.43** | **1, 16** | **0.022** |
|  | Replicate (2) | -0.128 | 0.493 | 0.07 | 1, 16 | 0.798 |
| *ADAMTS18* | Intercept | 2.521 | 0.439 |  |  |  |
|  | **Selection Regime (L)** | **-1.310** | **0.490** | **7.14** | **1, 15** | **0.017** |
|  | Replicate (2) | -0.041 | 0.490 | 0.01 | 1, 15 | 0.935 |
| *TLR3* | Intercept | 0.632 | 0.159 |  |  |  |
|  | **Selection Regime (L)** | **0.547** | **0.187** | **8.54** | **1, 16** | **0.010** |
|  | Replicate (2) | 0.253 | 0.187 | 1.83 | 1, 16 | 0.195 |
| *Mx* | Intercept | 3.032 | 0.473 |  |  |  |
|  | Selection Regime (L) | 1.127 | 0.556 | 4.11 | 1, 16 | 0.060 |
|  | Replicate (2) | -0.873 | 0.556 | 2.46 | 1, 16 | 0.136 |
| *ASPN* | Intercept | 1.593 | 0.309 |  |  |  |
|  | **Selection Regime (L)** | **1.072** | **0.345** | **9.65** | **1, 15** | **0.007** |
|  | Replicate (2) | 2.089 | 0.265 | 3.06 | 1, 15 | 0.101 |

**Table S6.**

Differences in follicle growth rate between the selection regimes. A difference in the growth rate of follicles between the lines is revealed by the significant selection regime*time interaction. Significance was determined by comparing nested models using likelihood ratio tests. Degrees of freedom are 1 in each case. Statistically significant effects are highlighted in bold. N = 5 follicles from 55 females each.

| Predictor | Estimate | SE | 𝛸^2^ | P |
| --- | --- | --- | --- | --- |
| *Fixed Effects* |  |  |  |  |
| Intercept | 1.016 | 0.364 |  |  |
| Time^a^ | 0.437 | 0.007 |  |  |
| Selection Regime (L) ^a^ | -0.115 | 0.027 |  |  |
| **Selection Regime * Time** | **-0.036** | **0.010** | **11.91** | **<0.001** |
| **Replicate (2)** | **0.487** | **0.225** | **4.62** | **0.032** |
| **Tarsus Length** | **0.022** | **0.009** | **6.02** | **0.014** |
| *Random Effects* |  |  |  |  |
| ID intercepts | 6.70 x 10^-3^ |  |  |  |
| ID slopes | 1.86 x 10^-6^ |  |  |  |
| Intercept-Slope Correlation | 0.33 |  |  |  |
| Residual | 2.18 x 10^-3^ |  |  |  |

^a^Test statistics are not presented because the interaction effect was significant

**Table S7.**

Differences in egg size and specific antibody response against a novel antigen (KLH) between the selection regimes in generation six and seven. Both the final model and full immune response model (i.e. with and without sex*regime interaction) are shown. Degrees of freedom are 1 in each case. Statistically significant effects are highlighted in bold.

| Trait | Predictor | Estimate | SE | 𝛸^2^ | *P* |
| --- | --- | --- | --- | --- | --- |
| Egg Size (N=73) | Intercept | 12.062 | 0.395 |  |  |
|  | **Selection Regime (L)** | **-1.145** | **0.245** | **19.46** | **<0.001** |
|  | Replicate (2) | -0.295 | 0.245 | 1.48 | 0.224 |
|  | Generation (7) | 0.257 | 0.370 | 0.53 | 0.467 |
| *Random Effects* | |  |  |  |  |
|  | Mother | 0.026 |  |  |  |
|  | Residual | 1.043 |  |  |  |
|  |  |  |  |  |  |
| Immune Response | Intercept | 0.190 | 0.070 |  |  |
| Final Model | **Selection Regime (L)** | **0.119** | **0.052** | **5.47** | **0.019** |
| (N=151) | Replicate (2) | -0.036 | 0.052 | 0.52 | 0.470 |
|  | Generation (7) | 0.023 | 0.064 | 0.14 | 0.711 |
|  | Sex (F) | -0.056 | 0.040 | 2.03 | 0.154 |
| *Random Effects* | |  |  |  |  |
|  | Mother | 0.015 |  |  |  |
|  | Residual | 0.054 |  |  |  |
|  |  |  |  |  |  |
| Immune Response | Intercept | 0.164 | 0.073 |  |  |
| Full Model | Selection Regime (L) | 0.173 | 0.064 |  |  |
| (N=151) | Replicate (2) | -0.039 | 0.052 |  |  |
|  | Generation (7) | 0.022 | 0.064 |  |  |
|  | Sex (F) | -0.001 | 0.057 |  |  |
|  | Selection Regime * Sex | -0.113 | 0.080 | 2.01 | 0.156 |
| *Random Effects* | |  |  |  |  |
|  | Mother | 0.016 |  |  |  |
|  | Residual | 0.053 |  |  |  |

**Table S8.**

Primers used for targeted quantitative PCR

| Target | Primers |
| --- | --- |
| *ADAMTS18* | 3’-TGTGGTTACAGACACCGTGC-5’  3’-TAACTTTTGCCCAGCCCCTT-5’ |
| *ASPN* | 3’-TTTTGCCAACATACCAAGCA-5’  3’-CGAATGAAGGAAGACCACCT-5’ |
| *KIAA1211* | 3’-TGTCTGCTTAGGAGGACTGGT-5’  3’-TGGCACTGAACGAGAGCAAA-5’ |
| *Mx* | 3’-GGGAACCAGCCACAAGATAA-5’  3’-TCCACCTCTTGAGCCATTTT-5’ |
| *NELL2* | 3’-GGCAGAATTATGCGGACAGT-5’  3’-AGGACCTGCACAATGAAAGG-5’ |
| *TLR3* | 3’-AACGAACCTTTCAAAACCCTGG-5’  3’-GCTGTACAACACAAGCTCACG-5’ |
| *VTG2* | 3’-CGCTGTATGAAAGGGTGCTC-5’  3’-TTGTCAGCCAGGTTGGAAGC-5’ |
| *β-actin* | 3’-CTGGCACCTAGCACAATGAA-5’  3’-CTGCTTGCTGATCCACATCT-5’ |

**Supplementary Figures**


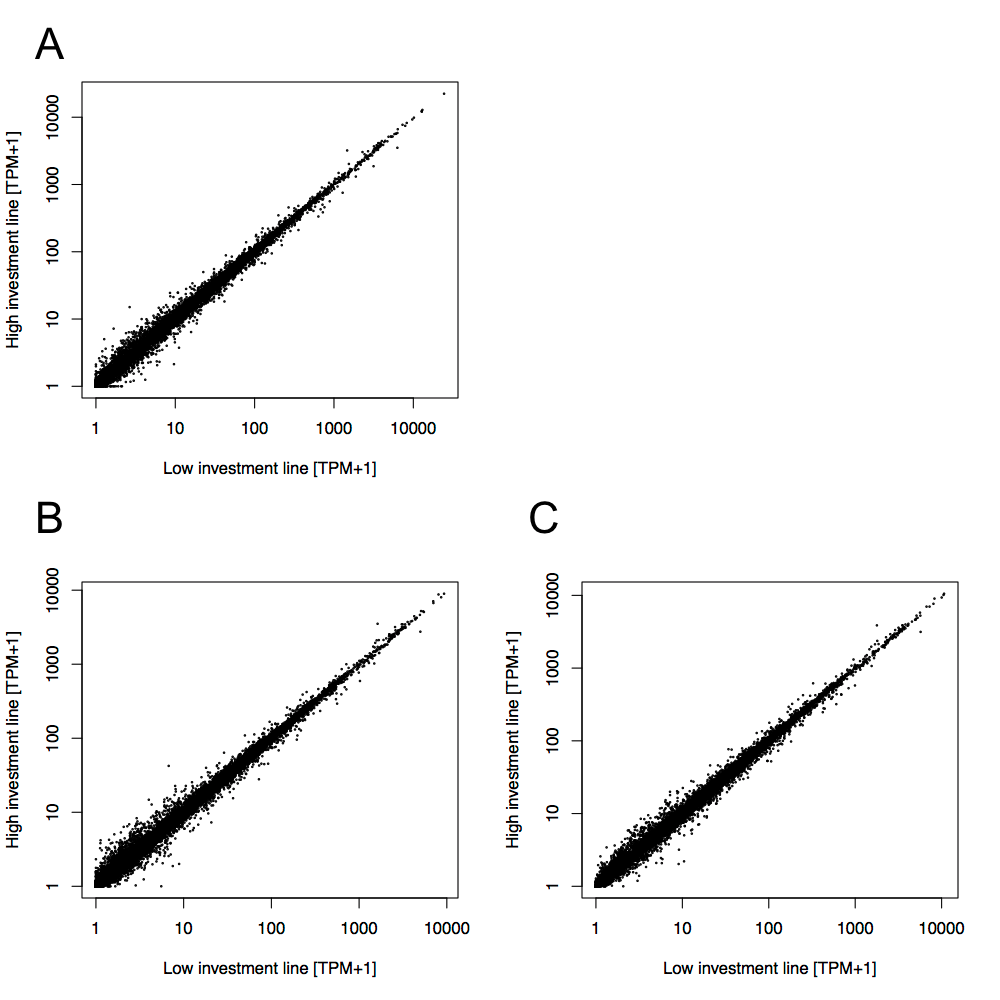
**Figure S1.**

Mean levels of gene expression (Transcripts Per Millions) between the high and low investment lines based on the A) chicken genome (*Gallus gallus*, Ensemble release 84), B) Coturnix japonica 2.0, C) Coja_2.0a. Each point corresponds to one gene.


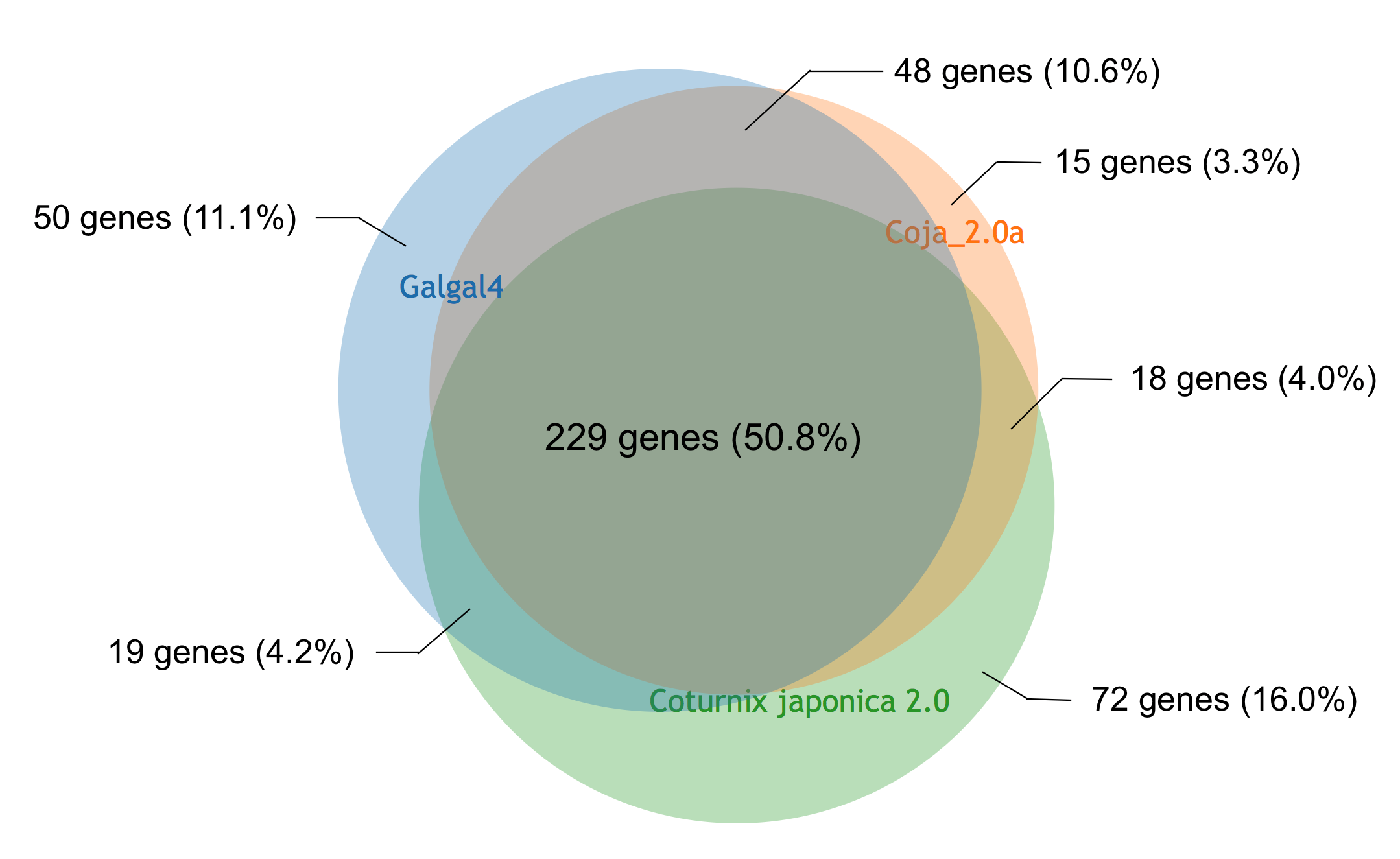
**Figure S2.**

Venn diagram showing the overlap of differentially expressed genes between different reference genomes. The size of circle and shared area are corresponding to the number of genes. The blue circle, green circle, and orange circle are corresponding to the chicken genome (*Gallus gallus*, Ensembl release 84), Coturnix japonica 2.0, and Coja_2.0a, respectively.
